# Supplementary material for: The implementation of the Workforce Indicators of Staffing Needs (WISN) method to improve access to health workforce in selected South-East Asian countries
Source: PLOS Glob Public Health. 2025 Jun 13;5(6):e0004727. doi: 10.1371/journal.pgph.0004727 (PMC12165366; doi:10.1371/journal.pgph.0004727)
Supplement: S1 Text — (PDF) [file pgph.0004727.s001.pdf]

Date of administration: **Ethics approval number:**

How can WISN be implemented to improve access to health workforce at the primary healthcare level?

Individual interview guide for policymakers and Primary Care Health Workers involved with the implementation of WISN

Thank you for agreeing to take time from your busy schedule to answer a few questions on our research. We are doing a study to find out more about the process involved to implement the Workload Indicators of Staffing Needs (WISN) in your country or primary care health facility where you work. The study aims to understand the implementation of the WISN for primary health care workforce planning in 11 countries of the World Health Organization South East Asia Region (WHO-SEAR).

You are being interviewed because you are a manager that was involved in some way with the WISN implementation process. The information collected will only be used to enrich the health workforce planning in primary care settings of many countries including yours. All the names from this discussion will be de-identified and your identified responses will not be shared outside the research team. The audio recording will be transcribed using Microsoft Teams. Your inputs are, therefore, highly valued. The aim of the research is not to assess professional competence and the outcomes of the research will not have a negative impact on your employment. You are welcome to refer to internal implementation documents to remind you of details that you might have forgotten. It’s also okay to not have all the answers. Please remember that the session is being audio recorded. You are welcome to let us know if you are not comfortable with that.

If you are happy with contents of this document and agree with the process, could you kindly sign the consent form and return to us? I am happy to answer any questions that you may have before we begin the discussion. Are there any questions that you would like to ask on the process before we start?

Date of administration: **Ethics approval number:**

No.	Area of Interest/topic	Initial broad descriptive questions	Possible probing questions
1	Professional Role in Country	Can you tell me more about your specific role within the Ministry of Health in your country?	What is your involvement in health workforce planning?<br>How did the country determine health workforce before WISN?
2	Involvement with WISN	Could you tell me about the pilot phase?<br>How was the WISN team set-up within the country?	How did you decide on pilot States/health facilities?<br>How long was the planned pilot period and \was it completed on schedule?
4	WISN planning	At what stage of planning were the WISN guidelines and standards for primary health care developed?<br>How were the packages of care required in primary care determined?<br>What consultation processes were undertaken and how were they undertaken?<br>How were the staff numbers and skills-mix required for efficient service delivery in primary care determined?	Who determined the approach used for implementation of WISN?<br>Could you tell us about the approach used (collaborative, top-down, bottom-up)?<br>What was the role of Primary Care service providers in the implementation process?<br>Were there changes introduced to the primary health care packages of care that resulted from the planned implementation of WISN?
5	WISN implementation	Could you tell me briefly about the strategy used to sample States/provinces, districts and primary health care facilities for the implementation of WISN.<br>What support (technical or financial) did you need to implement WISN?<br>Were some primary health care staff trained on WISN methods and/or how the software could be used?	Did you have access to the WISN user manual?<br>Was the WISN tool installed in all primary health care health facilities?<br>What was the interaction of primary health care health facilities with other arms of government when WISN was being implemented?
6	WISN Successes	What would you say are the successes of the WISN implementation process in your country?	Are there any visible successes or impacts that you can link with the introduction of WISN?
7	WISN Challenges	What challenges did your team experience during the implementation of WISN?	Were WISN determined staffing needs implemented in primary care facilities?
8	Overall results	How has your experience been using WISN for workforce planning?	

Wrap-up

Are the any other issues not covered that you would like to talk about?

Date of administration: **Ethics approval number:**

Thank you once more for your assistance, could you please also help me with a few documents that will help broaden my understanding on the schemes. You could add any other documents to the list if you think it or they will be of importance.
