# Supplementary material for: The implementation of the Workforce Indicators of Staffing Needs (WISN) method to improve access to health workforce in selected South-East Asian countries
Source: PLOS Glob Public Health. 2025 Jun 13;5(6):e0004727. doi: 10.1371/journal.pgph.0004727 (PMC12165366; doi:10.1371/journal.pgph.0004727)
Supplement: Attachment — Submitted filename: Reviewer Comments_SM1.docx [file pgph.0004727.s003.docx]

**REFERENCE:** **PLOS Global Public Health** **PGPH-D-24-01831**

**Title:** The implementation of the Workforce Indicators of Staffing Needs (WISN) method to improve access to health workforce in selected South-East Asian countries

**The Editor:**

**PLOS Global Public Health**

**RE:** **PGPH-D-24-01831**

Thank you for considering our manuscript, the comments from the reviewers have strengthened our manuscript and we have responded to all comments and questions in chronological order and stratified by reviewer.

Sincerely,

Prof. Sikhumbuzo A. Mabunda

**Reviewer #1:**

Dear authors,

Comments:

**Comment 1:** Congratulations for a well written article. You have done a very good job!

**Response:** Thank you.

**Comment 2:** I have very few recommendations as you done a great job:

**Response:** Thank you

**Comment 3:** In the introduction, you focus quite a lot on WISN, but do not provide very much context on the countries studied, which would allow the reader to better understand your results.

**Response:** Thank you for the comment. We have now added a statement in the Introduction (line 128-130) to give context that: “The countries of interest are all classified as low- and middle-income countries (LMICs) with high health workforce needs, thus requiring efficient health workforce planning strategies”.
**Comment 4:** It would be beneficial to present what was the reasoning for the choice of methodology.
**Response:** Thank you for the advice. Line 149-150 of the Methods now state that: “This strategy was chosen as it was the most practical, efficient and comprehensive strategy to ascertain the implementation of WISN in these six South East Asian countries”.

**Reviewer #2:**

**Abstract**

**Comment 1:** Although there are no requirements for separate subheadings, it will be useful to keep the balance.

**Response:** Thank you for the advice.

**Comment 2:** Please indicate the need for this research in one of the first sentences (before the methods).

**Response:** Thank you for the comment. We have added a sentence (Line 32-33) which states that: “This research ascertained whether WISN’s recommendations were adopted and operationalised after the initial assessment”.

**Comment 3:** It is stated “The Workload Indicators of Staffing Needs (WISN) is a tool used to estimate health workforce requirements for national or sub-national regions and health facilities. We determined the implementation of WISN for six countries in the World Health Organization South-East Asia Region (WHO-SEAR).” However, it is not clear why the research is crucial, its possible impact and/or the current gaps are.

**Response:** Thank you for the comment. A sentence (Line 33-35) which states that: “This will then help policymakers identify implementation barriers and enablers for the successful implementation of WISN for the improvement of access to health workforce”.
**Comment 4:** “We used a multi-method approach comprising qualitative, in-depth, semi-structured interviews, literature reviews and report or document reviews” – what do authors mean by “and report or document reviews”? Report analysis and document reviews?

**Response:** Thank you for the comment. The World Health Organization (WHO) had expectations for implementing countries to write reports that would be approved by WHO, either country, regional or global office. These are the reports being referred to. However, there are documents that countries generated internally, some not published or signed off. These also include planning and implementation documents.

To clear the confusion, we have since adopted your suggestion of referring to this methodological strategy merely as “…document reviews” (Line 36).
**Comment 5:** Additionally, the authors present information mainly through interviews with stakeholders (in-depth or semi-structured?) but no information on how various literature reviews were conducted.
**Response:** Thank you for the comment, the Methods section has added a statement (Line 40-42) to clarify that: “Literature reviews were conducted using electronic databases, documents and reports were sourced from the WHO-SEAR office and/or country of interest’s focal persons”.

**Comment 6:** On the other hand, “Transcription errors were corrected by the first author by listening to the audio and editing all identified Errors” is too much information and is better placed in the results section of the manuscript.
**Response:** Thank you for the comment. This has been removed in the Abstract but retained in the main Methods to ensure replication. This would be misplaced in the Results section.

**Comment 7:** Keywords -“WISN; Health workforce OR Human resources for health OR HRH AND Planning; Health systems” – the Boolean operators are redundant here. Please delete.
**Response:** Thank you for the comment. The advice has been implanted in full.

**Introduction:**

**Comment 8:** “Considering the need for efficient and effective health workforce management, the World Health Organization (WHO) developed the Workload Indicators of Staffing Needs (WISN), a method to calculate human resource requirements for health systems, considering the healthcare delivery models and population demands [5].” – needs restructuring, it is challenging to follow the logic here.

**Response:** Thank you for the comment. The sentence has now been broken down into two (Line 82-86). “Considering the need for efficient and effective health workforce management, the World Health Organization (WHO) developed the Workload Indicators of Staffing Needs (WISN), a method to calculate human resource requirements for health systems [5]. Unlike traditional techniques such as the population ratio methods, WISN considers the healthcare delivery models and population demands [5]”.
**Comment 9:** Although it is generally clear that this study is needed, it is worth elaborating on particular (health) related problems in South Asia. Why is there acute health workforce shortage and what does it mean for South Asia (perhaps elaboration on the most prominent diseases, public health issues, etc).

**Response:** Thank you for the comment. We have considered the advice and added a paragraph on the burden of diseases, health worker shortages and their reasons in South East Asia. The paragraph (Line 114-121) reads:

“After sub-Saharan Africa, South-East Asia has some of the most acute health workforce shortages in the world[14]. This is also a region where demographic and epidemiological changes are occurring at a fast pace and the need for a trained and available health workforce is crucial. For instance, even though these countries are LMICs, noncommunicable diseases (NCDs) caused nearly two thirds of all deaths in WHO South-East Asian countries[15]. It is also important noting that this region is home to over a quarter of the world’s population, which shares a disproportionate burden of disease and mortality [16]. It is projected that this region will have 40% of the global health workforce shortages that are estimated for 2030 [17]. These shortages are mostly attributed to maldistribution, emigration of the health workforce, substandard working conditions, poor planning, training challenges, gaps in health workforce data, and weak health workforce governance[17]”.

**Comment 10:** Have there been any attempts to clarify staffing needs in the past? (possibly comparable to Workload Indicators of Staffing Needs (WISN) assessment).

**Response:** Thank you for the comment. This has now been clarified (Line 130-131) to state that: “There are literature gaps on the use of WISN recommendations beyond the initial determination of the human resources for health gaps as was the case in South Africa [21]”.
**Comment 11:** What is the aim/objective of this research?

**Response:** Thank you for the comment. The Introduction (Line 125-128) now clarifies to state that: “Therefore, this study aimed to ascertain the implementation of the Workload Indicators of Staffing Needs (WISN) recommendations for primary health care health workforce planning for countries in the World Health Organization South East Asia Region (WHO-SEAR)”.

**Comment 12:** Are there any research questions the authors want to address?
**Response**: Thank you for the comment. We hope that the expansion of the aims and significance of the study now helps clarify the study objectives further (Line 128-136): “The countries of interest are all classified as low- and middle-income countries (LMICs) with high health workforce needs, thus requiring efficient health workforce planning strategies. There are literature gaps on the use of WISN recommendations beyond the initial determination of the human resources for health gaps as was the case in South Africa [21]. This study will therefore help to determine whether WISN’s recommendations were adopted after the initial assessments in countries in the WHO South-East Asia region. It is then hoped that these findings will help policymakers identify implementation barriers and enablers for the successful implementation of WISN for the improvement of access to health workforce in the countries studied, those who have used WISN and those planning to use it in future”.

**Materials and Methods:**

**Comment 13:** The authors are suggested to re-write the methods section and add relevant references for the data collection methods used in this study (multi-methods approach). The section needs extensive revision.

**Response:** Thank you for the advice. This section has been edited and appropriately referenced.
**Comment 14:** Why/when semi-structured and/or in-depth interviews?

**Response:** Thank you for the comment. Line 146-148 of the Methods states that: “…we conducted in-depth interviews of policymakers between 17 June 2022 and 30 August 2022 to understand the implementation of WISN in the respective countries”. Line 149-150 states that: This strategy was chosen as it was the most practical, efficient and comprehensive strategy to ascertain the implementation of WISN recommendations in these six South East Asian countries.

**Comment 15:** Regarding the literature and report reviews, are there any publication date limits? Language?

**Response:** Thank you for the comment. No, there were no language or date restrictions, merely because this was only restricted to the 6/11 countries under the World Health Organization South-East Asia region. We have since edited a statement (Line 143-144) to reflect that all the reviews had no language or date restrictions…

**Comment 16:** Why this review methods were preferred?

**Response:** Thank you for the comment. A paragraph has been added (Line 151-154) to reflect that: Literature reviews were conducted using electronic databases (PUBMED, EBSCO-HOST, Google Scholar and Google), documents and reports were sourced from the WHO-SEAR office and/or country of interest’s focal persons who were either employed by the Ministry of Health or WHO. This review strategy was used to ensure that all relevant studies (whether published or not) would be included.

**Comment 17:** Also the authors reveal some results in the methods section “Five interviews from four countries were conducted virtually by RJ using Microsoft Teams and lasted an average of 47 minutes. The research team did not manage to get into contact with an Indonesian and Nepalese focal people for WISN using multiple strategies. Two participants were interviewed separately for Bhutan” – please keep the methods consistent.

**Response:** Thank you for the comment. Because this study also uses Qualitative Research Methods, it is important to have an audit trail to ensure rigor of the findings. The quoted statements are therefore part of the Methods but not Results as they explain what was done to obtain the findings.

**Comment 18:**“Data were audio-recorded and transcribed using Microsoft Teams after obtaining consent from participant” – this information appeared earlier in the manuscript.
**Response:** Thank you for the comment. The statement in line 187-188 has been deleted to avoid duplication.

**Comment 19:** “The last Table in the Results section summarises reflections on the process and the utility of WISN by the five interviewed participants from four of the countries” – What is the number of the table you are referring to?

**Response:** Thank you for the comment. The statement has since been deleted.
**Comment 20a:** Sampling and, Identification of Literature and Reports – it is unclear if there were any inclusion or exclusion criteria.

**Response:** Line 168-170 of the Methods states that: Medline, CINAHL, Google and Google Scholar searches were conducted to identify **all** literature on WISN from these six WHO South East Asian countries. There were therefore no exclusion criteria as long as studies were on WISN, they were included.

**Comment 20b:** Although sampling bias are not avoidable, please reveal essential characteristics of involved stakeholders.

**Response:** Thank you for the comment. Line 152-153 states that: “…documents and reports were sourced from the WHO-SEAR office and/or country of interest’s focal persons who were either employed by the Ministry of Health or WHO”.

**Comment 21:** “Medline and CINAHL searches were conducted to identify all literature on WISN from these five WHO South East Asian countries” – dates/limits? Why did the authors use only scientific databases? Especially when there is a statement of applying report reviews?

**Response:** Thank you for the comment. The previous statement had omitted the use of Google and Google Scholar, this has now been incorporated in two places in the document (Line 151-152 and Line 168-169).
**Comment 22:** “Role of the funding source This work was funded by WHO SEARO. Data collection, analysis and interpretation was led by researchers in consultation with country-based researchers and policymakers.” – it seems not appropriate in the methods section of the manuscript
**Response:** Thank you for the comment. The journal editorial previously requested us to include this component within the Methods section. We are happy to remove it if that is the new requirement.

**Results**

**Comment 23:** It is not completely clear how the multi-methods approach was triangulated.

**Response:** Thank you for the comment. Line 197-199 of the Methods section states that: “The results synthesised and triangulated the findings of the reports and interviews and presented them by country as there were different variables and approaches in each country”.

Results from the three methods were integrated and presented as if they are from one source but are stratified by country. Results sourced from literature and reports/documents are then cited to acknowledge the source.

**Comment 24:** How did the authors compensate for the lack of data from the stakeholder interviews in Indonesia and Nepal?
**Response:** Thank you for the comment. A limitation has been added (Line 560-562) to state that: “Even though the reports and literature for Indonesia and Nepal were comprehensive, they could not completely compensate for the lack of interviews”.

**Comment 25:** Additionally, there is some inconsistency in data presentation across the countries: for example, there is an overview (table 4) Short, medium and long-term WISN recommendations in Bhutan, this is the only country that provides this.

**Response:** Thank you for the comment. The three themes used for the presentation of results are “…overview and initial implementation, key findings, primary lessons and directions forward”. In this way we are then able to present an unbiased view of the process of implementation of WISN from beginning to end. A statement of clarity (Line 201) has been incorporated to reflect that: “These themes focus on the process flow of WISN implementation”. Furthermore, these themes have been incorporated within the results. The cited example from Table 4 therefore refers to key findings that Bhutan reported. It is difficult to therefore have such comparisons between countries if those are not the recommendations made for that country. A limitation has also been added in line 562-565 reflecting that: “…despite attempts to ensure comparability of findings and recommendations between countries, this was not always the case. However, this study was able to achieve its objective of ascertaining whether the recommendations were utilised beyond the WISN determinations”.
**Comment 27:** Overall, there is inconsistency in the presentation of all six countries, which makes it difficult for the reader to understand why the analysis are not comparable. The authors claim to use semi-structured and in-depth interviews using the interview guide. Additionally the review analysis could support the consistency of data presentation (themes).
Bangladesh: Workload indicator staffing needs ratio and health workforce requirements for Bangladesh.
Bhutan: Short, medium and long-term WISN recommendations in Bhutan.
India: WISN Ratio results for four cadres in two Indian health facilities
Indonesia: WISN ratios of the various cadres included in Indonesia
Nepal: Nepal's process flow on the implementation of WISN; Health workforce staffing needs for various cadres in Nepal; Short and long-term recommendations (without a table like in Bhutan).
Sri Lanka: Sri Lanka’s WISN ratio for Public Health Midwives (PHMs)

**Response:** Thank you for the comment. We hope that incorporation of the themes within the text and the clarity provided above helps to strengthen the manuscript.

**Comment 28:** “Reflections from four of the countries are summarised in Table 7” – it is worth mentioning that the opinions from the stakeholders are based on one interview per country (!) (with an exception of 2 in Bhutan), so the authors should be careful with the statement “Reflections from four of the countries” – instead those are the single opinions.
General for the results: although the majority of the findings are exciting and informative, the authors should restructure the section, especially for single countries.

**Response:** Thank you for the comment and advice. This has been corrected and now is: “Reflections from key informants of four of the countries”.

**Comment 29:** The tables and figures are diverse, covering various aspects. For example, for Sri Lanka there is a table for WISN ratio for Public Health Midwives, it is particular profession.

**Response:** Thank you for the comment. The comment on the diversity of the Tables and Figures is noted and has been addressed elsewhere in this document.

**Comment 30:** Also, it is not clear whether PH is considered to be a part of health workforce in the country. If so, please elaborate on the relevance of PH workforce (and its definition) in the introduction.

**Response:** Thank you for the comment. Line 97-101 of the introduction has now incorporated a referenced statement which states that: “Furthermore, the utility of WISN is not only limited to the determination of skilled health professionals (e.g., physicians, nurses and midwives) but it can be extended to be used in the determination of public health cadres (e.g., community health workers, volunteers, etc.) and midlevel workers who are the backbone of primary health care provision in many LMICs”.
**Comment 31:** Generally, it is challenging to go through separate county information (sic); please help the reader to structure the information (to enhance the generalizability of the findings).
**Response:** Thank you for the comment, this is appreciated and acknowledged. Our hope is that policymakers and the WHO will look at country specific findings and easily locate differences and similarities between countries in approaches and solutions. It is therefore for this reason that we have chosen to retain the separate country reporting. We do however, hope that incorporation of the themes will help improve clarity and flow of the results.
**Discussion:**

**Comment 32:** I believe that WISN could be successfully used to understand staffing needs. However, it is essential to understand the limitations of this study's design. Only four out of six countries provide insights from single interviews, making eliminating subjective opinions extremely challenging.
Also, there could be an interplay and definition clash between the health workforce and the PH workforce (e.g., PH midwives).
The authors present recommendations (general for all 6 countries?); how does the reader deal with the recommendations provided by representatives from Bhutan and Nepal? Could you please clarify the generalizability issues (transferability of the results)?
**Response:** Thank you for the comment. The authors have now clarified the study’s aim to be focused on ascertaining the use of WISN recommendations in the different countries. This then means that the only measure of comparability is on the use of the recommendations and not necessarily how WISN was implemented. We hope that the added limitations (Line 560-565) and the refined aim provides clarity on this: “Even though the reports and literature for Indonesia and Nepal were comprehensive, they could not completely compensate for the lack of interviews. Furthermore, despite attempts to ensure comparability of findings and recommendations between countries, this was not always the case. However, this study was able to achieve its objective of ascertaining whether the recommendations were utilised beyond the WISN determinations”.

**General comments:**
**Comment 33:** There is confusion about the use of Level 1-2-3 heading throughout the manuscript (e.g. the results are the same level heading as subsections within it)

**Response:** Thank you for the comment. We have followed the journal’s guidelines on the font and hope that the Journal’s editing will help eliminate any incongruent fonts or styles.
